# Comparing Gly^11^/dAla^11^-Replacement vs. the in-Situ Neprilysin-Inhibition Approach on the Tumor-targeting Efficacy of the ^111^In-SB3/^111^In-SB4 Radiotracer Pair

**DOI:** 10.3390/molecules24061015

**Published:** 2019-03-13

**Authors:** Emmanouil Lymperis, Aikaterini Kaloudi, Panagiotis Kanellopoulos, Marion de Jong, Eric P. Krenning, Berthold A. Nock, Theodosia Maina

**Affiliations:** 1 Molecular Radiopharmacy, INRASTES, NCSR “Demokritos”, 15310 Athens, Greece; mlymperis@hotmail.com (E.L.); katerinakaloudi@yahoo.gr (A.K.); kanelospan@gmail.com (P.K.); nock_berthold.a@hotmail.com (B.A.N.); 2 Department of Radiology, Erasmus MC, 3015 GD Rotterdam, The Netherlands; m.hendriks-dejong@erasmusmc.nl; 3 Cytrotron Rotterdam BV, Erasmus MC, 3015 GD Rotterdam, The Netherlands; erickrenning@gmail.com

**Keywords:** GRPR-antagonist, bombesin-like radioligand, tumor targeting, tumor imaging, neprilysin-inhibition, phosphoramidon, in vivo stability

## Abstract

*Background*: The GRPR-antagonist ^68^Ga-SB3 visualized prostate cancer lesions in animal models and in patients. Switching radiometal from ^68^Ga to ^111^In impaired tumor targeting in mice, but coinjection of the neprilysin (NEP)-inhibitor phosphoramidon (PA) stabilized ^111^In-SB3 in circulation and remarkably increased tumor uptake. We herein report on the biological profile of ^111^In-SB4: ^111^In-[dAla^11^]SB3. *Methods*: The biological responses of ^111^In-SB3/SB4 were compared in PC-3 cells and animal models. *Results*: Gly^11^/dAla^11^-replacement deteriorated GRPR-affinity (SB4 IC_50_: 10.7 ± 0.9 nM vs. SB3 IC_50_: 4.6 ± 0.3 nM) and uptake in PC-3 cells (^111^In-SB4: 1.3 ± 0.4% vs. ^111^In-SB3 16.2 ± 0.8% at 1 h). ^111^In-SB4 was more stable than ^111^In-SB3, but PA-coinjection stabilized both radiotracers in peripheral mice blood. Unmodified ^111^In-SB3 showed higher uptake in PC-3 xenografts (8.8 ± 3.0%ID/g) vs. ^111^In-SB4 (3.1 ± 1.1%ID/g) at 4 h pi. PA-coinjection improved tumor uptake, with ^111^In-SB3 still showing superior tumor targeting (38.3 ± 7.9%ID/g vs. 7.4 ± 0.3%ID/g for ^111^In-SB4). *Conclusions*: Replacement of Gly^11^ by dAla^11^ improved in vivo stability, however, at the cost of GRPR-affinity and cell uptake, eventually translating into inferior tumor uptake of ^111^In-SB4 vs. unmodified ^111^In-SB3. On the other hand, in-situ NEP-inhibition turned out to be a more efficient and direct strategy to optimize the in vivo profile of ^111^In-SB3, and potentially other peptide radiotracers.

## 1. Introduction

The gastrin-releasing peptide receptor (GRPR) has attracted much attention in cancer diagnosis and therapy [1,2,3,4]. High levels of GRPR expression have been documented in a variety of human cancers, including prostate, breast and lung cancer, as opposed to lack of expression in healthy surrounding tissue [5,6,7,8,9,10]. This combination renders GRPR an appealing biomolecular target for directing diagnostic/therapeutic radionuclide-carriers specifically onto cancer lesions in a personalized theranostic approach. Based on the GRPR natural ligands, bombesin (BBN), gastrin-releasing peptide (GRP) and its C-terminal fragments (e.g., GRP(17–27) or neuromedin C, NMC), several peptide analogs have been developed by strategic structural interventions to allow for stable binding of the desired radionuclide and aim at biological profiles promising for clinical translation [4]. Yet, such agonist-based radioligands activate the GRPR upon binding, eliciting side effects after injection to patients. Recently a shift of paradigm has occurred toward radiolabeled GRPR-antagonists that, besides their higher inherent biosafety, have unexpectedly shown superior tumor targeting and faster background clearance from the body compared to agonists [11,12]. 

**Figure 1:**
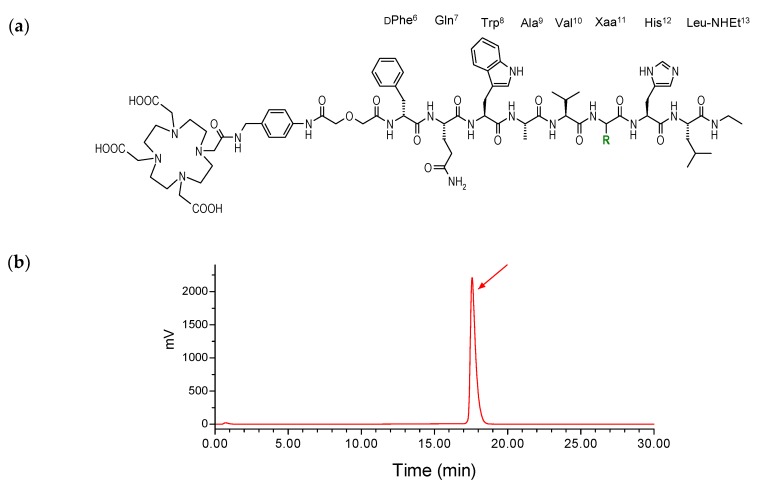
(**a**) Chemical structure of SB3 (Xaa^11^ = Gly, R = H) and SB4 (Xaa^11^ = dAla, R = CH_3_); (**b**) Typical radiochromatogram of HPLC analysis of ^111^In-SB4 labeling reaction mixture, showing quantitative formation of the high purity radioligand eluting at *t_R_* = 17.6 min (HPLC system 1).

As a part of our work on GRPR-directed theranostic agents, we have developed a series of radiolabeled analogs based on the potent GRPR-radioantagonist [dPhe^6^,Leu^13^-NHEt]BBN(6-13) [11,13,14,15]. Accordingly, SB3 was generated by coupling the universal DOTA chelator to its N-terminal dPhe^6^ via a suitable linker (SB3 = DOTA-*p*-aminomethylaniline-diglycolic acid-dPhe-Gln-Trp-Ala-Val-Gly-His-Leu-NHEt; Figure 1a), thereby allowing for labeling with SPECT (^111^In), PET (^68^Ga) and beta-emitting (^177^Lu) radionuclides for eventual theranostic clinical use [16,17,18]. The ^68^Ga-SB3 radiotracer in particular, was successful in visualizing 50% of prostate and breast cancer lesions in patients with advanced disease applying PET/CT [16]. In a following study including 10 therapy-naïve primary prostate cancer patients, lesion visualization with ^68^Ga-SB3 was successful in 90% of the cases. Most importantly, a significant correlation could be established between the intensity of uptake in the lesions and the GRPR-expression levels [19]. Surprisingly however, the theranostic prospects with SB3 were found compromised when switching radiometal from ^68^Ga to ^111^In or ^177^Lu. In preclinical studies, ^111^In/^177^Lu-SB3 displayed drastically impaired tumor localization levels compared to ^68^Ga-SB3, a result attributed to impaired metabolic stability of the ^111^In/^177^Lu-radiopeptides in mice. This result could be banned by coinjection of the neprilysin (NEP)-inhibitor phosphoramidon (PA), that stabilized ^111^In/^177^Lu-SB3 in peripheral mouse blood and remarkably improved tumor uptake [17]. 

Earlier work on the development of GRPR-antagonists generated a solid body of structure-activity relationships data, pinpointing at important factors for sustained in vivo action [12,20]. Metabolic stability was found to be a key prerequisite for sufficient delivery to GRPR-expressing cells and prolonged in vivo responses. Metabolic stability could be enhanced by suitable structural modifications, including dAla^11^/Gly^11^ in BBN-based or dAla^24^/Gly^24^ in GRP-based peptide ligands [20,21]. Interestingly, we made similar observations in a series of ^99m^Tc-labeled [dPhe^6^,Leu^13^-NHEt]BBN(6-13) analogs, carrying an acyclic tetraamine chelator at the N-terminus. Thus, ^99m^Tc-[N4-*p*-aminomethylaniline-diglycolic acid-dPhe^6^,dAla^11^,Leu^13^-NHEt]BBN(6-13) displayed the highest metabolic stability within this small library of compounds [22]. 

In the present work we have compared the two stabilization strategies, namely the structural dAla^11^/Gly^11^-substitution in the peptide sequence vs. the in situ NEP-inhibition approach [23], for their efficacy to improve tumor uptake and overall pharmacokinetics of resulting ^111^In-radiotracers in mice. For this purpose, we first developed SB4, [dAla^11^]SB3, and directly compared the in vitro and in vivo behavior of ^111^In-SB4 vs. ^111^In-SB3 in the same experimental models. We next studied the impact of NEP-inhibition after PA-coinjection on the in vivo stability and the tumor targeting capabilities of the two ^111^In-radiotracers. 

## 2. Results

### 2.1. Peptides and Radioligands

The new SB4 peptide conjugate was generated by single Gly^11^/dAla^11^-replacement in the SB3 peptide chain (Figure 1a). Both SB3 and SB4 carrying the universal chelator DOTA (1,4,7,10-tetraazacyclododecane-1,4,7,10-tetraacetic acid) at their N-terminus could be labeled with ^111^In at molecular activities of 3.7–7.4 MBq ^111^In/nmol peptide. Quality control by radioanalytical HPLC demonstrated in all cases labeling yields >98% in a >96% radiochemical purity. Hence, the resultant ^111^In-SB3 and ^111^In-SB4 radioligands were used without further purification in all subsequent experiments. A representative radiochromatogram of analysis of labeling reaction product ^111^In-SB4 is included in Figure 1b. 

### 2.2. In Vitro Assays in PC-3 Cells

#### 2.2.1. Affinity of SB3 and SB4 for the GRPR

**Figure 2:**
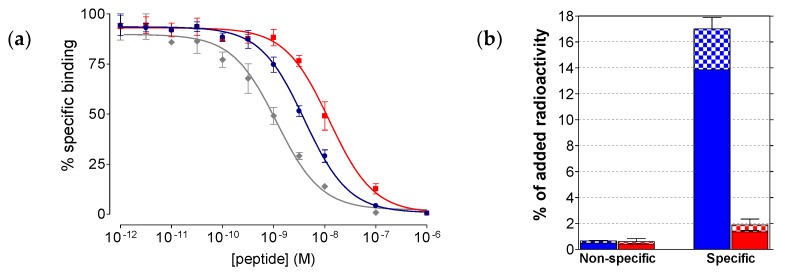
(**a**) [^125^I-Tyr^4^]BBN displacement curves from GRPR-sites on PC-3 cells after 1 h incubation at 22 °C by ■ SB4 (IC_50_ 11.2 ± 1.1 nM, *n* = 3), ● SB3 (IC_50_ 4.6 ± 0.3 nM, *n* = 3) and ◆ [Tyr^4^]BBN (IC_50_ 1.3 ± 0.1 nM, *n* = 5); (**b**) Non-specific and GRPR-specific association of ^111^In-SB3 (blue) and ^111^In-SB4 (red) in PC-3 cells after 1 h incubation at 37 °C. Results represent average cell associated activity ± sd (solid bars: membrane bound; checkered bars: internalized) vs. total-added activity (*n* = 4, in triplicate); non-specific values were obtained in the presence of 1 μM [Tyr^4^]BBN and were subtracted from totals to provide the specific values; the study was conducted with PC-3 cells as confluent monolayers.

As shown in Figure 2a, SB3, SB4 as well as the [Tyr^4^]BBN reference were able to displace [^125^I-Tyr^4^]BBN from GRPR-sites on PC-3 cell membranes in a monophasic and dose-dependent manner. The respective half-maximal inhibitory concentration (IC_50_) values differed, yielding the following rank of increasing receptor affinity: SB4 (IC_50_ = 11.2 ± 1.1 nM, *n* = 3) < SB3 (IC_50_ = 4.6 ± 0.3 nM, *n* = 3) < [Tyr^4^]BBN (IC_50_ = 1.3 ± 0.1 nM, *n* = 5). Hence, single Gly^11^/dAla^11^-replacement in the SB3 peptide chain caused a significant drop of binding affinity of SB4 to GRPR (*p* < 0.001). 

#### 2.2.2. Comparative Uptake of ^111^In-SB3 and ^111^In-SB4 by PC-3 Cells

During 1 h incubation at 37 °C in PC-3 cells, ^111^In-SB3 and ^111^In-SB4 were taken up by the cells via a GRPR-mediated process, as demonstrated by the drop of cell uptake in the presence of excess [Tyr^4^]BBN (Figure 2b). In both cases, the bulk of radioactivity remained bound to the cell-membrane with only a small portion internalizing into the cells, as consistent with a radioantagonist profile [11]. However, ^111^In-SB4 showed much lower overall uptake in PC-3 cells (1.9 ± 0.5% of total added activity) vs. ^111^In-SB3 (16.2 ± 0.8% of total added activity; *p* < 0.0001), further demonstrating the negative impact of the Gly^11^/dAla^11^-substitution on the interaction ability of the forming radiotracer with the GRPR. 

### 2.3. In Vivo Comparison of ^111^In-SB3 and ^111^In-SB4

#### 2.3.1. Stability of ^111^In-SB3 and ^111^In-SB4 in Healthy Mice

**Figure 3:**
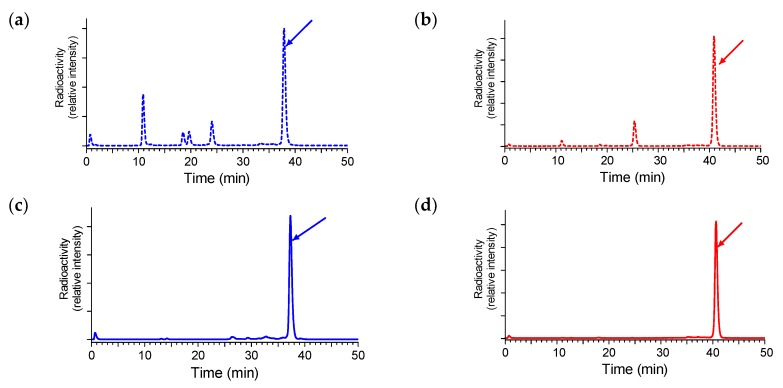
Radiochromatograms of HPLC analysis of mouse blood samples collected 5 min pi, of (**a**) ^111^In-SB3 (55% intact radiotracer) or (**b**) ^111^In-SB4 (77% intact radiotracer) without PA-coinjection; the respective radiochromatograms of (**c**) ^111^In-SB3 (98.9% intact radiotracer), or (**d**) ^111^In-SB4 (99.7% intact radiotracer) with PA-coinjection are also included; the *t*_R_ of parent radiopeptide was determined by coinjection with the respective radioligand sample in the column (HPLC system 2) and is indicated here by the arrow.

The two ^111^In-SB3 and ^111^In-SB4 radiotracers exhibited different metabolic stability in peripheral mouse blood. As revealed by HPLC analysis of blood samples collected from mice at 5 min after radioligand injection, the dAla^11^-substituted ^111^In-SB4 was markedly more stable (80 ± 3% intact, *n* = 3) than non-modified ^111^In-SB3 (56 ± 2% intact, *n* = 3; *p* < 0.001), revealing the positive influence of the adopted structural intervention on metabolic stability; representative radiochromatograms for ^111^In-SB3 and ^111^In-SB4 are shown in Figure 3a,b, respectively. 

It is interesting to note that coinjection of the NEP-inhibitor PA stabilized both ^111^In-SB3 and ^111^In-SB4 in mouse circulation (Figure 3c,d, respectively; *p* < 0.001), revealing NEP as the major degrading protease in vivo. Hence, the in-situ NEP-inhibition strategy turned out to be more efficacious in metabolically stabilizing the radioligand than the structural modification approach, pursued herein via Gly^11^/dAla^11^-replacement. 

#### 2.3.2. Comparative Biodistribution of ^111^In-SB3 and ^111^In-SB4 in SCID Mice Bearing PC-3 Xenografts

**Figure 4:**
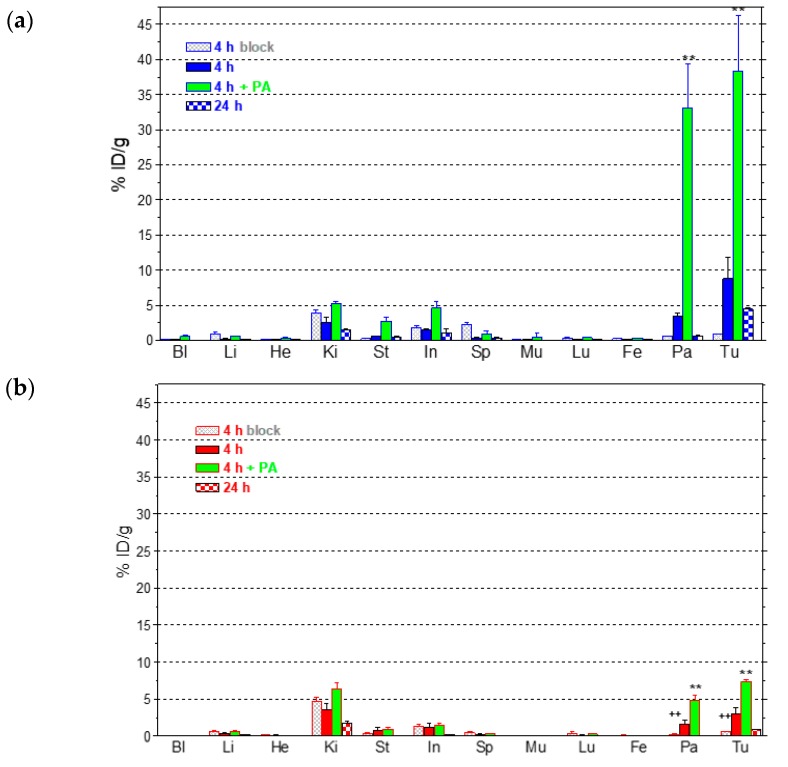
Biodistribution data for (**a**) ^111^In-SB3 or (**b**) ^111^In-SB4 in SCID mice bearing subcutaneous PC-3 xenografts in their flanks at 4 and 24 h pi; two additional animal groups at the 4 h pi interval comprise mice coinjected with excess [Tyr^4^]BBN for GRPR-blockade (block) or a 300 µg dose of PA for in situ inhibition of NEP (PA). Data is expressed as average ± sd %ID/g, *n* = 4 and plots were drawn in the same scale of uptake for easy comparison; statistically significant differences are indicated by ** or ++ between controls and block or PA groups, respectively, at 4 h pi, as estimated by one-way ANOVA with Tukey’s post-hoc analysis. Bl = blood, Li = liver, He = heart, Ki = kidneys, St = stomach, In = intestine, Sp = spleen, Mu = muscle, Lu = lungs, Fe = femur, Pa = pancreas and Tu = PC-3 tumor.

The biodistribution of ^111^In-SB3 and ^111^In-SB4 was studied in severe combined immune deficiency (SCID) mice bearing human PC-3 xenografts expressing the GRPR. Subcutaneous tumors of suitable size developed in the flanks of mice about four weeks after inoculation of a suspension of prostate adenocarcinoma PC-3 cells and biodistribution was conducted. Comparative tissue distribution results, expressed as percent injected dose per gram (%ID/g) and presented as average %ID/g ± sd (*n* = 4), are included in Figure 4a for ^111^In-SB3 and Figure 4b for ^111^In-SB4. 

Both tracers showed rapid blood clearance with quite similar distribution patterns in all non-GRPR-expressing tissues at all time points. The radioactivity cleared from the body of mice via the kidneys and the urinary tract. However, significant differences in the uptake of the two tracers were evident in the PC-3 tumors and the tissues physiologically expressing the GRPR, such as the pancreas. Thus, tumor uptake of non-modified ^111^In-SB3 were 8.8 ± 3.0%ID/g at 4 h pi declining to 4.4 ± 0.3%ID/g at 24 h pi, whereas the respective values for ^111^In-SB4 were 3.0 ± 0.8%ID/g (*p* < 0.01) and 0.9 ± 0.1%ID/g (*p* < 0.0001). Likewise, ^111^In-SB3 exhibited higher pancreatic uptake compared to ^111^In-SB4 both at 4 h pi (3.5 ± 0.4%ID/g and 1.6 ± 0.8%ID/g, respectively; *p* < 0.01) and at 24 h (0.6 ± 0.1%ID/g and 0.1 ± 0.0%ID/g, respectively; *p* < 0.001). It should be noted that tumor and pancreas values achieved by ^111^In-SB4 at 4 h pi were significantly reduced after coinjection of excess [Tyr^4^]BBN, suggesting a GRPR-mediated uptake (*p* < 0.001). For ^111^In-SB3 however, no significant change was found adopting the one way ANOVA test with Tukey’s post-hoc analysis due to the wide variance of values and the unpaired two tailed Student’s t test was additionally conducted revealing highly significant differences (*p* < 0.001). 

The effect of metabolic stabilization of both radiotracers during NEP-inhibition was studied in separate animal groups co-injected with a 300 µg dose of PA. As a result, the tumor uptake was markedly improved for both radioligands, but was most affected in the case of unmodified ^111^In-SB3 compared to the more in vivo robust ^111^In-SB4. Thus, in the case of ^111^In-SB3 a 4.3-fold enhancement of tumor uptake was accomplished at 4 h pi by PA coinjection (38.3 ± 7.9%ID/g – PA - vs. 8.8 ± 3.0%ID/g – control; *p* < 0.001) with only 2.4-fold improvement achieved for ^111^In-SB4 (7.3 ± 0.3%ID/g – PA – vs. 3.0 ± 0.8%ID/g – control; *p* < 0.001). Likewise, higher enhancement was observed in the uptake of ^111^In-SB3 compared to ^111^In-SB4 after PA-coinjection in all tissues with physiological GRPR-expression, especially in the mouse pancreas (33.1 ± 6.2%ID/g vs. 4.9 ± 0.7%ID/g, respectively; *p* < 0.001). 

## 3. Discussion

Radiotracers based on GRPR-antagonists have been lately attracting much attention in nuclear medicine compared to agonists, largely because of their higher inherent biosafety [11,12]. First radioligands developed for nuclear medicine purposes were based on linear native BBN-/GRP-sequences, which, following systemic administration and subsequent GRPR-binding, internalized into target cells while at the same time activating the receptor and eliciting adverse effects [2,3,4]. In contrast, radiolabeled GRPR-antagonists, that neither internalize nor activate the GRPR upon binding, turned out to be more appropriate for human use [20]. Moreover, recent studies have shown that radiolabeled GRPR-antagonists unexpectedly achieved superior tumor targeting and better pharmacokinetics compared to their agonist counterparts [12]. It should be noted that GRPR-antagonists are actually synthetic compounds designed to inhibit the action of agonists in the body, preferably for long periods of time. Accordingly, they are tailored to better resist in vivo degradation through strategic chemical changes of their structure, as for example by introduction of unnatural amino acids, reduction or methylation of amide bonds and other means [20]. It is therefore, reasonable to assume that radiolabeled GRPR-antagonists for nuclear medicine use would also be more stable than agonists. 

Following this rationale, we have been exploring the impact of in vivo metabolic stability on the biological profile of radiolabeled GRPR-antagonists developed by our group. In one set of compounds, suitable chelators were coupled at the N-terminal of the potent GRPR-antagonist [dPhe^6^,Leu^13^-NHEt]BBN(6-13) via different linkers allowing for labeling with ^99m^Tc, ^68^Ga, ^111^In and ^177^Lu [15,16,22]. As expected, the resultant radiotracer uptake in PC-3 xenografts in mice was found to depend on in vivo stability. The latter was influenced by structural changes of the radiotracer not only on the peptide chain, but also the linker or the radiometal chelate. For example, in a small library of ^99m^Tc-DB1 mimics [22], the dAla^11^-substituted analog was the most in vivo robust member, in agreement with the higher stability reported for similarly substituted dAla^11^/Gly^11^-BBN-like or dAla^24^/Gly^24^-GRP-like analogs [21]. In another example, it was the radiometal/radiometal-chelate affecting in vivo stability and tumor uptake. Thus, by switching radiometal from ^68^Ga to ^111^In (or ^177^Lu) in SB3 we observed a drastic drop of metabolic stability translating into impaired targeting of ^111^In-SB3 PC-3 tumors in mice compared to ^68^Ga-SB3 [17,18]. 

In an effort to identify the major protease(s) involved in the fast in vivo degradation of radiolabeled [dPhe^6^,Leu^13^-NHEt]BBN(6-13) analogs, we have co-injected specific protease-inhibitors along with the radiopeptide and applied HPLC analysis to monitor potential changes in radiometabolite patterns induced in mouse blood [17,23]. Of great significance is the finding that coinjection of the NEP-inhibitor PA stabilized most GRPR-radioantagonists in vivo, leading to remarkable enhancement of radiolabel uptake in the implanted PC-3 tumors. The above results established NEP as a leading cause of in vivo catabolism of studied radioligands. Furthermore, the innovative concept of enhancing tumor targeting via in-situ altering the immediate milieu of the radiopeptide on its way to the target instead of modifying its structure warrants further investigation. 

In the present study we first introduced SB4, a single dAla^11^/Gly^11^-substution derivative of SB3. This modification was shown to enhance radiotracer stability without greatly impairing other biological features (e.g. receptor affinity or in vivo tumor targeting) in a series of ^99m^Tc-DB1 mimics [22]. In the present study however, we observed a ≈2.5-fold drop of affinity to the GRPR by comparing SB3 (IC_50_ = 4.6 ± 0.3 nM, n=3) to SB4 (IC_50_ = 11.2 ± 1.1 nM, *n* = 3; *p* < 0.001). We have not compared the receptor affinities of the In-metalated species, given that the type and position of the forming metal-chelate was identical in the two analogs, which differed only at position 11 of the peptide chain. Furthermore, the overall specific uptake of the respective ^111^In-radioltracers in PC-3 cells after 1 h incubation at 37 ^o^C significantly dropped from 16.2 ± 0.8% (^111^In-SB3) to 1.9 ± 0.5% (^111^In-SB4) of total added activity (*p* < 0.0001). This drop followed the same trend of receptor affinity deterioration observed in the unlabeled species and further corroborated the negative effect of dAla^11^/Gly^11^-substution on the in vitro interaction of SB4/^111^In-SB4 with the GRPR (Figure 2). However, the in vivo stability of ^111^In-SB4 in peripheral mouse blood (80 ± 3% intact, *n* = 3) was found significantly increased compared to ^111^In-SB3 (56 ± 2% intact, *n* = 3; *p* < 0.001) (Figure 3). It is interesting to note that the pursued dAla^11^/Gly^11^-replacement, albeit improving in vivo stability, negatively influenced the interaction with the GRPR, eventually translating into poorer uptake of ^111^In-SB4 in PC-3 tumors in mice (Figure 4). At both the 4 h and the 24 h pi time intervals unmodified ^111^In-SB3 (8.8 ± 3.0%ID/g and 4.4 ± 0.3%ID/g, respectively) showed superior uptake compared to ^111^In-SB4 (3.0 ± 0.8%ID/g and 0.9 ± 0.1%ID/g, respectively; *p* < 0.01), revealing the negative impact of this modification on the overall performance of resulting radiotracer. This unfavorable result was found to be surprisingly pronounced in the ^111^In-SB3/^111^In-SB4 pair compared to what was expected from previous observations on other analogs [21] and highlights the fact that extrapolation of structural changes across molecules may lead to different or even opposite effects. 

Aiming at assessing the impact of the alternative NEP-inhibition approach on the in vivo performance of ^111^In-SB3 and ^111^In-SB4, we have co-injected PA and studied the changes induced on the in vivo stability and tumor uptake of both radioligands at 4 h pi. Firstly, we observed an impressive full stabilization of both radiotracers in mice blood (Figure 3). Most importantly, this stabilization translated into remarkably enhanced tumor values for both non-modified ^111^In-SB3 (38.3 ± 7.9%ID/g; *p* < 0.001) and the dAla^11^/Gly^11^-modified ^111^In-SB4 (7.3 ± 0.3%ID/g; *p* < 0.001). It is clearly evident that in-situ NEP-inhibition is a direct and more effective strategy than structural intervention to improve tumor targeting in this pair of GRPR-radioantagonists. 

However, several issues need to be addressed first before proposing this exciting approach for clinical application of GRPR-radioantagonists or of a broader spectrum of peptide analogs. Thus, protease-inhibitor administration as such or in combination with the radiopeptide in question has to be proven safe for patients. Next, the increased costs of extended toxicology tests and production of GMP-grade compounds for clinical testing should be taken into account. Eventually, it is expected that approval by ethical committees will become more challenging when injection of combinations of substances is planned. On the other hand, the in situ protease-inhibition concept, once clinically established for a certain radiopeptide, may be more easily extrapolated thereafter to a broader spectrum of radiopeptide candidates for clinical translation. Undoubtedly, this methodology provides a powerful preclinical tool to: (i) identify enzymes truly involved in the degradation of biodegradable peptide radioligands, (ii) reveal cleavage sites and (iii) indicate the range of feasible improvements for a particular tumor-target model induced by radiopeptide stabilization. 

## 4. Materials and Methods

### 4.1. Peptides and Radioligands

The SB3 (DOTA-*p*-aminomethylaniline-diglycolic acid-dPhe-Gln-Trp-Ala-Val-Gly-His- Leu-NHEt) and SB4 (DOTA-*p*-aminomethylaniline-diglycolic acid-dPhe-Gln-Trp-Ala-Val-dAla-His- Leu-NHEt; Figure 1) peptide conjugates were synthesized on the solid support as previously reported [17] and were provided by PiChem (Graz, Austria). The [Tyr^4^]BBN (Tyr^4^-bombesin, Pyr-Gln-Arg-Tyr-Gly-Asn-Gln-Trp-Ala-Val-Gly-His-Leu-Met-NH_2_) reference was purchased from PSL GmbH (Heidelberg, Germany). 

Radioiodination of [Tyr^4^]BBN was performed using ^125^I ([^125^I]NaI in 0.1 N NaOH (pH 12–14) provided by Perkin Elmer) according to the chloramine-T methodology, as previously described [22]. The forming sulfoxide (Met^14^=O) was reduced by dithiothreitol and [^125^I-Tyr^4^]BBN was isolated in non-carrier added form by HPLC. Methionine was added to the purified radioligand solution to prevent re-oxidation of Met^14^ to the corresponding sulfoxide and the resulting stock solution in 0.1% BSA-PBS was kept at −20 °C; aliquots thereof were used in competition binding assays (molar activity of 2.2 Ci/μmol). 

#### Preparation and Quality Control of ^111^In-SB3 and ^111^In-SB4

Lyophilized SB3 or SB4 was dissolved in HPLC-grade H_2_O (2 mg/mL) and 50 μL aliquots thereof were stored in Eppendorf Protein LoBind tubes at −20 °C. For labeling, ^111^InCl_3_ in 50 mM HC1 (activity concentration of 0.37 GBq/mL on calibration date) was employed (Mallinckrodt Medical B.V., Petten, The Netherlands). In Eppendorf Protein LoBind tubes (1.5 mL capacity) ^111^InCl_3_ solution (150 µL, 55–110 MBq) was mixed with SB3 or SB4 (15 nmol), 1 M sodium acetate buffer pH 4.6 (20 µL) was added and the mixture was left to react at 85 °C for 20 min. For in vivo studies ^111^In-SB3 and ^111^In-SB4 were obtained at molar activities of 3.7–7.4 MBq ^111^In/nmol DOTA-conjugate. 

Reversed-phase HPLC was performed on a Waters Chromatograph based on a 600E multisolvent delivery system coupled to a Waters 2998 photodiode array detector and a Gabi gamma-detector (Raytest, RSM Analytische Instrumente GmbH). Data processing and chromatography were controlled by the Empower Software (Waters, Vienna, Austria). For quality control 2 μL aliquots of the radiolabeling solution were quenched with 28 μL of an acetate buffered solution of DTPA (1 mM, pH 4.6) and a Symmetry Shield RP18 cartridge column (5 μm, 3.9 mm × 20 mm, Waters) was used for analyses. Solutes were eluted with 0.1% TFA/MeCN applying a linear gradient starting from 0% MeCN and a 2% increase per min at 1 mL/min flow rate (system 1). The radiochemical labeling yield exceeded 98% and the radiochemical purity was >99%. Samples of ^111^In-SB3 and ^111^In-SB4 were analyzed before and after completion of all biological experiments. 

### 4.2. In Vitro Assays

#### 4.2.1. Cell Lines and Culture

Human androgen-independent prostate adenocarcinoma PC-3 cells endogenously expressing the human GRPR (LGC Promochem, Teddington, UK) were used in the present study [24]. Cells were cultured in Roswell Park Memorial Institute (RPMI)-1640 medium, supplemented with 10% heat-inactivated fetal bovine serum (FBS), 100 U/mL penicillin and 100 µg/mL streptomycin, and kept in a controlled humidified atmosphere containing 5% CO_2_ at 37 °C. Passages were performed weekly using a trypsin/EDTA (0.05%/0.02% *w*/*v*) solution. All culture media were purchased from Gibco BRL, Life Technologies and supplements were provided by Biochrom KG Seromed. 

#### 4.2.2. Competition Binding in PC-3 Cell-Membranes

Competition binding experiments against [^125^I-Tyr^4^]BBN were performed with SB3, SB4, or [Tyr^4^]-BBN (reference) in PC-3 cell membranes, prepared as previously reported [25]. For the assay, triplicates per concentration point (concentration range: 10^−13^–10^−6^ M) of each test peptide were incubated together with the radioligand (~40,000 cpm per assay tube, at a 50 pM concentration) in PC-3 cell-membrane homogenates in a total volume of 300 μL binding buffer (BB, 50 mM HEPES pH 7.4, 1% BSA, 5.5 mM MgCl_2_, 35 μM bacitracin) for 1 h at 22 °C in an Incubator-Orbital Shaker (MPM Instr. SrI, Italy). Binding was interrupted by ice-cold washing buffer (WB, 10 mM HEPES pH 7.4, 150 mM NaCl) and rapid filtration (Whatman GF/B filters presoaked in BB) on a Brandel Cell Harvester (Adi Hassel Ing. Büro, Munich, Germany). Filters were washed with ice-cold WB and counted in an automatic well-type gamma counter (NaI(Tl) 3´´-crystal, Cobra Packard Auto-Gamma 5000 series instrument). The half maximal inhibitory concentration (IC_50_) values were calculated using nonlinear regression according to a one-site model applying the PRISM 2 program (Graph Pad Software, San Diego, CA) and represent the mean±sd from three independent experiments performed in triplicate. 

#### 4.2.3. Internalization Assay in PC-3 Cells

The overall cell association – internalization of ^111^In-SB3 and ^111^In-SB4 was assessed in PC-3 cells. Briefly, PC-3 cells were seeded in six-well plates (~1 × 10^6^ cells per well) 24 h before the experiment. Approximately 50,000 cpm of either ^111^In-SB3 or ^111^In-SB4 (corresponding to 250 fmol total peptide in 150 μL of 0.5% BSA/PBS) was added alone (total) or in the presence of 1 μM [Tyr^4^]BBN (non-specific). Cells were incubated at 37 °C for 1 h and incubation was interrupted by placing the plates on ice, removing the supernatants and rapid rinsing with ice-cold 0.5% BSA/PBS. Cells were then treated 2 × 5 min with acid wash buffer (2 × 0.6 mL, 50 mM glycine buffer pH 2.8, 0.1 M NaCl) at room temperature and supernatants were collected (membrane-bound fraction). After rinsing with 1 mL chilled 0.5% BSA/PBS, cells were lyzed by treatment with 1 N NaOH (2 × 0.6 mL) and lysates were collected (internalized fraction). Sample radioactivity was measured in the γ-counter and total cell-associated (internalized+membrane bound) radioactivity was determined vs. total added activity. Results represent the average values ± sd of four experiments performed in triplicate. 

### 4.3. Animal Studies

#### 4.3.1. In Vivo Stability Tests

For stability experiments (approved protocol # 1609, Prefecture of Attica), healthy male Swiss albino mice (30 ± 5 g, NCSR “Demokritos” Animal House Facility) were used. The radioligand, ^111^In-SB3 or ^111^In-SB4, was injected as a 100 μL bolus (11–22 MBq, 3 nmol total peptide) in the tail vein together with injection solution (100 µL; control) or with a phosphoramidon (PA)-solution (100 µL injection solution containing 300 µg PA). Animals were euthanized and blood (0.5–1 mL) was directly withdrawn from the heart and transferred in a pre-chilled EDTA-containing Eppendorf tube on ice. Blood samples were centrifuged for 10 min at 2000 g/4 °C and plasma was collected. After addition of an equal volume of ice-cold MeCN the mixture was centrifuged for 10 min at 15,000 g/4 °C. The supernatant was concentrated under a N_2_-flux at 40 °C to 0.05-0.1 mL, diluted with saline (0.4 mL), filtered through a 0.22 μm Millex GV filter (Millipore, Milford, USA) and analyzed by RP-HPLC. The Symmetry Shield RP18 (5 μm, 3.9 mm × 20 mm) column was eluted at a flow rate of 1.0 mL/min with the following linear gradient (system 2): 0% B at 0 min to 10% B in 10 min and then in 40 min to 30% B; A = 20 mM ammonium acetate and B = MeCN. The *t*_R_ of the intact radiopeptide was determined by coinjection with the ^111^In-SB3 or ^111^In-SB4 reference in the HPLC. 

#### 4.3.2. Induction of PC-3 Xenografts in SCID Mice

A suspension containing freshly harvested human PC-3 cells (≈150 μL of a ≈1.2 × 10^7^ cells) was subcutaneously injected in the flanks of female SCID mice (15 ± 3 g, six weeks of age at the day of arrival, NCSR “Demokritos” Animal House Facility). The animals were kept under aseptic conditions and 4 weeks later developed well-palpable tumors (80–200 mg) at the inoculation sites (approved protocol # 1610, Prefecture of Attica). 

#### 4.3.3. Biodistribution in PC-3 Xenograft-Bearing SCID Mice

For the biodistribution study (approved protocol # 1610, Prefecture of Attica), animals in groups of 4 received via the tail vein a 100 μL bolus of ^111^In-SB3 or ^111^In-SB4 (37 kBq, corresponding to 10 pmol total peptide) co-injected either with injection solution (100 μL; control) or PA-solution (300 μg PA dissolved in 100 μL injection solution; 4 h + PA), or with excess [Tyr^4^]BBN (100 μL injection solution containing 50 μg [Tyr^4^]BBN for in vivo GRPR-blockade; 4 h block). Animals were euthanized at 4 and 24 h pi and dissected; samples of blood, tumors and organs of interest were collected, weighed and measured for radioactivity in the gamma counter. Intestines and stomach were not emptied of their contents. Data was calculated as percent injected dose per gram tissue (%ID/g) with the aid of standard solutions and represent mean values±sd, *n* = 4. 

### 4.4. Statistical Analyses

The unpaired two tailed Student’s t test or one-way ANOVA with Tukey’s post-hoc analysis of GraphPad Prism Software (San Diego, CA) was applied to determine statistically significant differences. *p* values of <0.05 were considered to be statistically significant.
